# Supplementary material for: The chromatin remodeling factor ISW-1 integrates organismal responses against nuclear and mitochondrial stress
Source: Nat Commun. 2017 Nov 28;8:1818. doi: 10.1038/s41467-017-01903-8 (PMC5703887; doi:10.1038/s41467-017-01903-8)
Supplement: Supplementary file 1 — Supplementary information [file 41467_2017_1903_MOESM1_ESM.pdf]

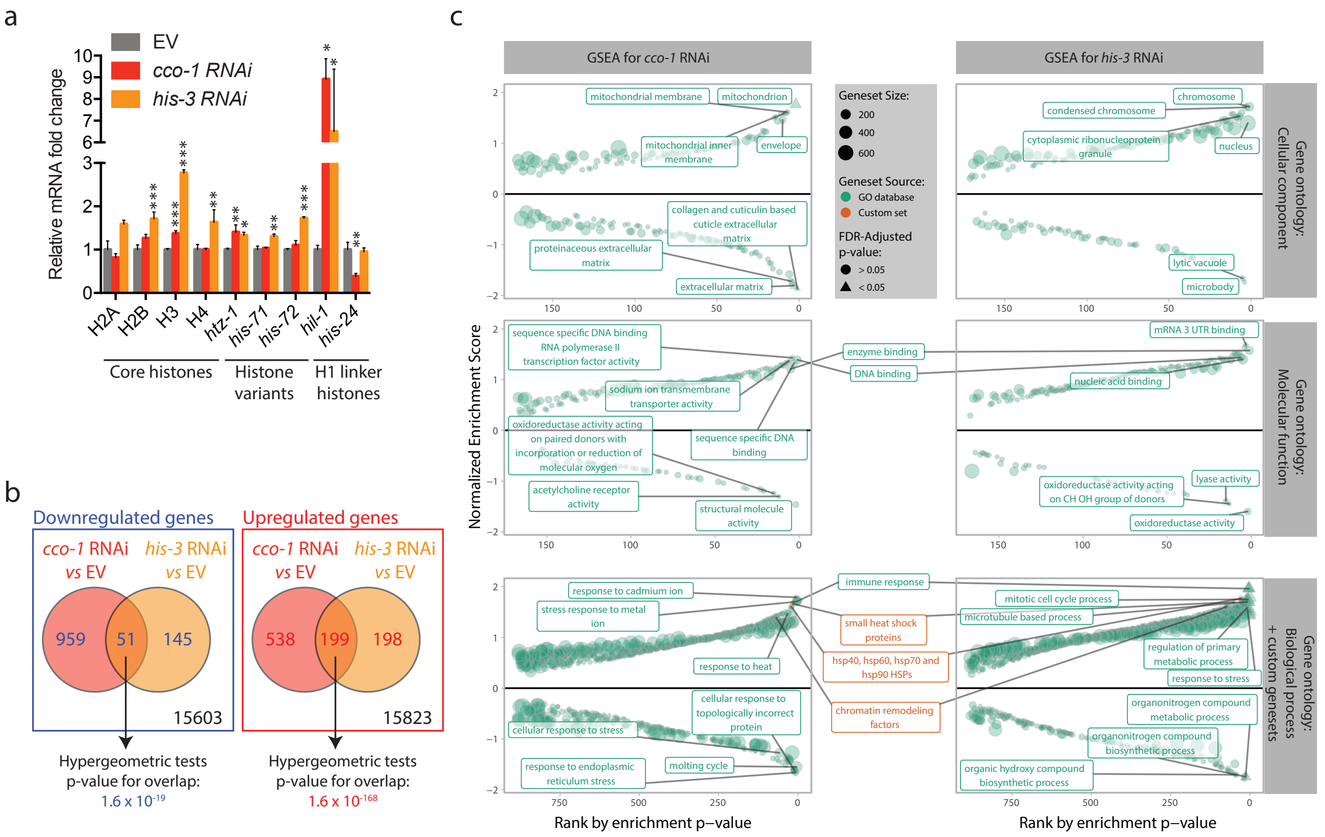

Supplementary Figure 1 Mitochondrial- and histone stress induce partly common changes in gene expression. (**a**) Histone gene expression after *cco-1* and *his-3* RNAi measured with qRT-qPCR. The H2A primer pairs have full sequence alignment with *his-12*, *his-16*, *his-30*, *his-33* and *his-43*, H2B pairs with *his-11*, *his-15*, *his-29*, *his-34* and *his-44*, H3 pairs with his-2, his-6, his-27, his-49 and his-40 (predicted pseudogene) and H4 pairs with *his-5*, *his-18*, *his-28*, *his-38* and *his-50*. N2 worms were collected for qRT-PCR analysis at L4 stage. Bars represent mRNA levels relative to empty vector with error bars indicating mean ± s.d. of three biological replicates, each with three technical replicates (**P*<0.05, ***P*<0.01, ****P*<0.001; one-way ANOVA with Dunnett's post test). (**b**) Venn diagram from RNA-seq data depicting commonly up- and downregulated genes between the treatments. (**c**) Gene set enrichment analysis results for the two treatment effects. Each point represents a gene set. Gene sets are ranked by increasing nominal p-value on the x-axis. The y-axis is the normalized enrichment score. Positive and negative enrichment scores indicate enrichment in up- and down-regulated genes, respectively. Custom genesets (see Supplementary Table 1) are colored orange. The size of the point represents the size of the gene set. Triangles represent gene sets that have an FDR-corrected p-value lower than 0.05.

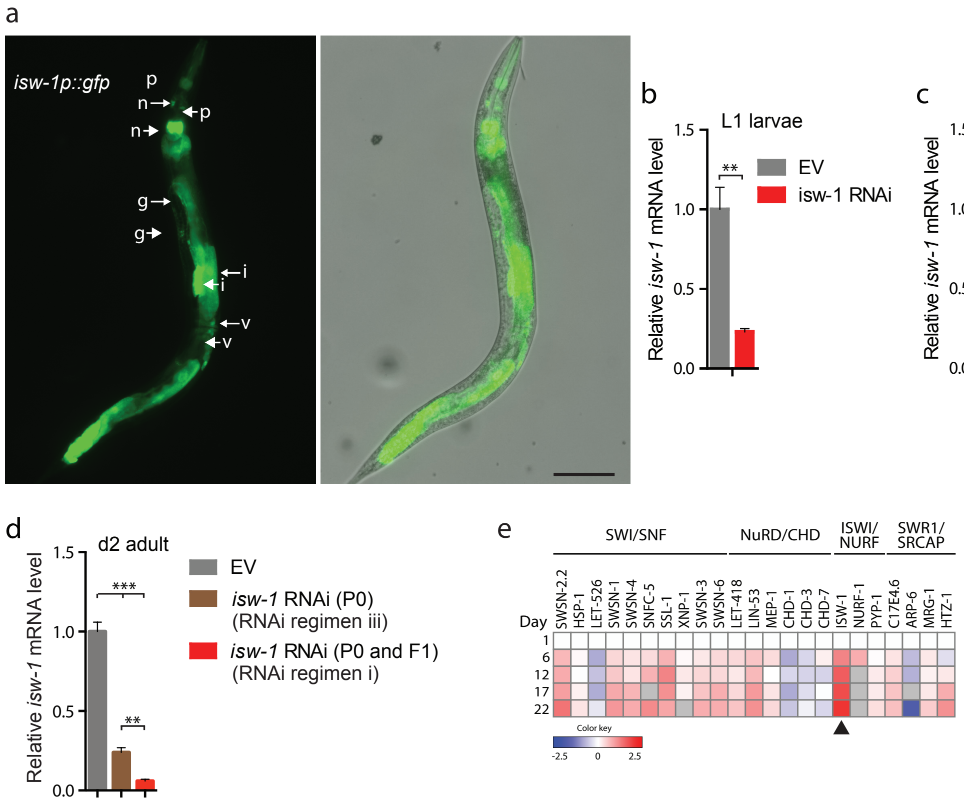

Supplementary Figure 2 *isw-1* expression profile, the effect of different RNAi regimens on on *isw-1* expression, and ISW-1 protein level upon ageing. (**a**) L4 larvae of wild-type worm expressing extrachromosomal array of *isw-1p::gfp* transgene. Abbreviations: p=pharynx, n=neurons, g=gonad, i=intestine, v=vulva. N = 5. Scale bar, 100 µm. (**b**) *isw-1* expression in L1 larvae of F1 generation after P0 generation was treated with EV or *isw-1* RNAi. Bars represent *isw-1* mRNA level relative to empty vector with error bars indicating mean ± s.d. of three biological replicates, each with three technical replicates (***P*<0.01; unpaired Student's *t*-test). (**c**) *isw-1* expression in L4 larvae of F1 generation after RNAi regimen i or ii. (**d**) *isw-1* expression in day 2 adult worms treated with *isw-1* RNAi by using RNAi regimen i or iii. In (**c**) and (**d**), bars represent *isw-1* mRNA level relative empty vector with error bars indicating mean ± s.d. of three biological replicates, each with three technical replicates (***P<0.01*, ****P*<0.001; one-way ANOVA with Tukey's post test). (**e**) Heatmap showing the protein levels of different chromatin remodeling factors in aging *C. elegans* using extant data set[24]. Colors indicate the fold change relative to day 0 (L4 larval stage). Arrow indicates the ISW-1, whose level is strongly increased upon aging.

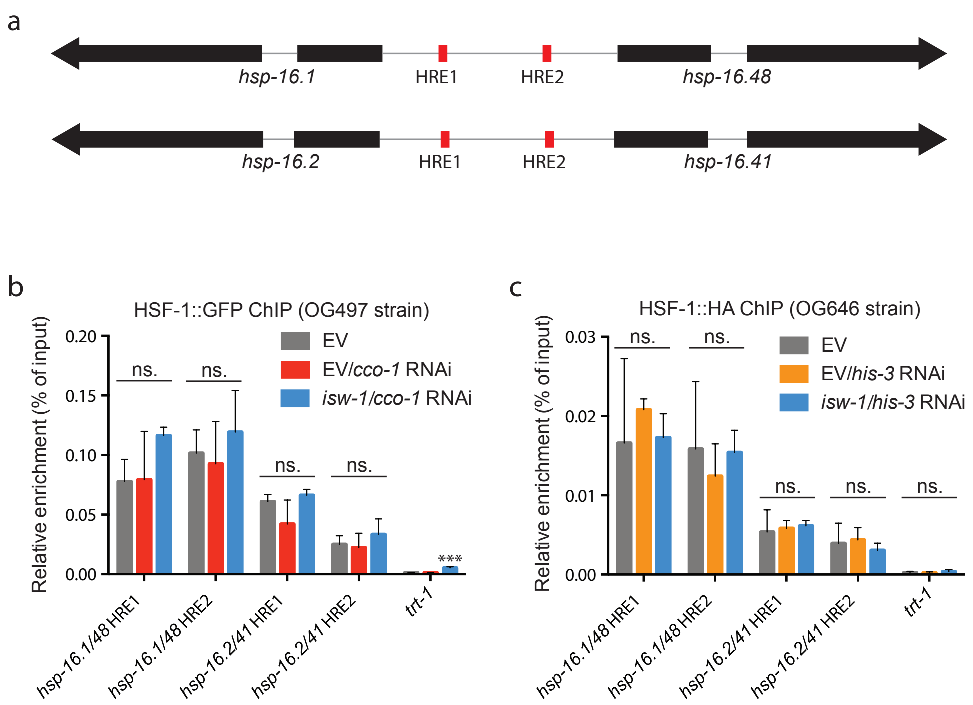

Supplementary Figure 3 *isw-1* RNAi does not affect HSF-1 binding on sHSP promoters. (**a**) Arrangement of *hsp-16* sHSP promoters and location of heat shock response elements (HRE). (**b**) HSF-1::GFP binding on HREs upon treatment with EV or combined EV/*cco-1* or *isw-1*/*cco-1* RNAi. (**c**) HSF-1::HA binding on HREs upon treatment with EV or combined EV/*his-3* or *isw-1*/*his-3* RNAi. In (**b**) and (**c**), measurement of HSF-1 binding on telomerase (*trt-1*) promoter, which does not contain HREs, was used as a control. Bars represent enrichment relative to input with error bars indicating mean ± s.d. of three biological replicates, each with three technical replicates (*$P<0.05$, **$P<0.01$, ***$P<0.001$; one-way ANOVA with Tukey's post test).

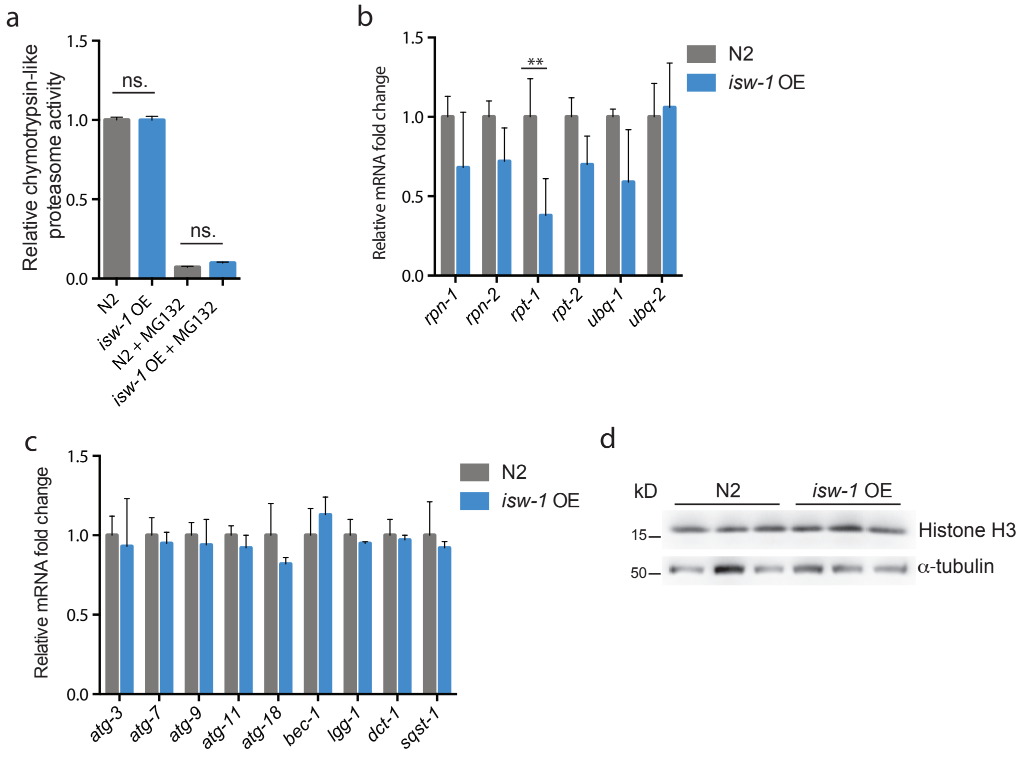

Supplementary Figure 4 *isw-1* OE strain phenotype is likely independent of proteasome and autophagy. (**a**) The proteasome activity in *isw-1* OE strain. Worms were collected for proteasome activity assay at day 2 of adulthood. Chymotrypsin-like proteasome activity was measured by using Suc-LLVY-AMC fluorescent peptide. Results shown are from one biological replicate. Bars represent peptide cleavage relative to N2 with error bars indicating mean ± s.d. of three technical replicates (one-way ANOVA with Tukey's post test). (**b**) The expression of proteasomal 19S genes (*rpn-1*, *rpn-2*, *rpt-1* and *rpt-2*) and ubiquitin (*ubq-1* and *ubq-2*) in N2 and *isw-1* OE strain. (**c**) The expression of autophagy and mitophagy genes in N2 and *isw-1* OE strain. In (**b**) and (**c**), worms were collected for qRT-PCR analysis at L4 stage. Bars represent mRNA levels relative to N2 with error bars indicating mean ± s.d. of three biological replicates, each with three technical replicates (**$P<0.01$; unpaired Student's *t*-test). (**d**) Histone H3 Western blots from N2 and *isw-1* OE worms. Results shown are from three biological replicates.

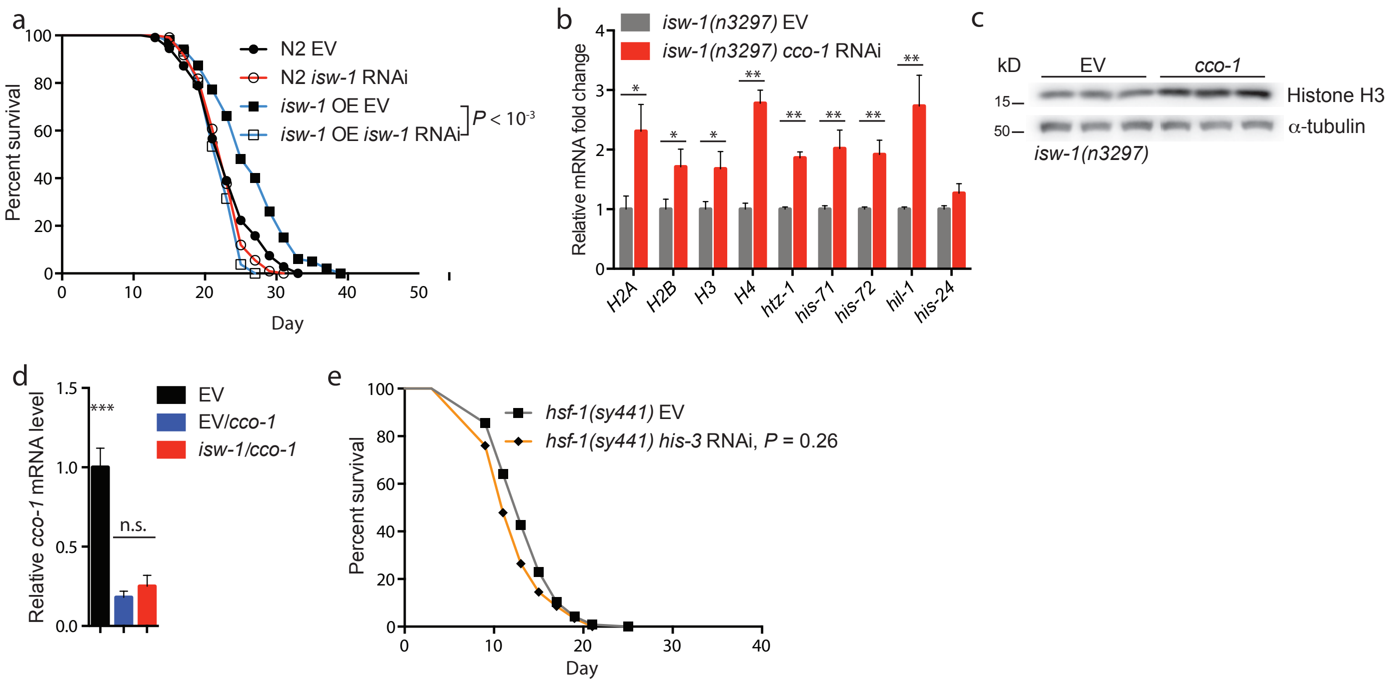

Supplementary Figure 5 ISW-1 regulates lifespan, but not histone levels. (**a**) Lifespan of N2 and *isw-1* OE strain on *isw-1* RNAi. (**b**) Histone gene expression in *isw-1(n3297)* mutants after *cco-1* RNAi measured with qRT-PCR. The H2A primer pairs have full sequence alignment with *his-12*, *his-16*, *his-30*, *his-33* and *his-43*, H2B pairs with *his-11*, *his-15*, *his-29*, *his-34* and *his-44*, H3 pairs with his-2, his-6, his-27, his-49 and his-40 (predicted pseudogene) and H4 pairs with *his-5*, *his-18*, *his-28*, *his-38* and *his-50*. Worms were collected for qRT-PCR analysis at L4 stage. Bars represent mRNA levels relative to empty vector with error bars indicating mean ± s.d. of three biological replicates, each with three technical replicates (**P*<0.05, ***P*<0.01; unpaired Student's *t*-test). (**c**) Histone H3 Western blots from *isw-1(n3297)* mutants after treatment with *cco-1* RNAi. Results shown are from three biological replicates. (**d**) *isw-1* knockdown does not affect the *cco-1* knockdown efficiency. RNAi treatment was initiated within L1 larvae of the generation used for qRT-PCR analysis (RNAi regimen ii, see Fig. 3a). N2 worms were collected for qRT-PCR analysis at L4 stage. Bars represent *cco-1* mRNA level relative to empty vector with error bars indicating mean ± s.d. of three biological replicates, each with three technical replicates (****P*<0.001; one-way ANOVA with Tukey's post test). (**e**) Lifespan of *hsf-1(sy441)* on *his-3* RNAi. See Supplementary table 2 for lifespan statistics.

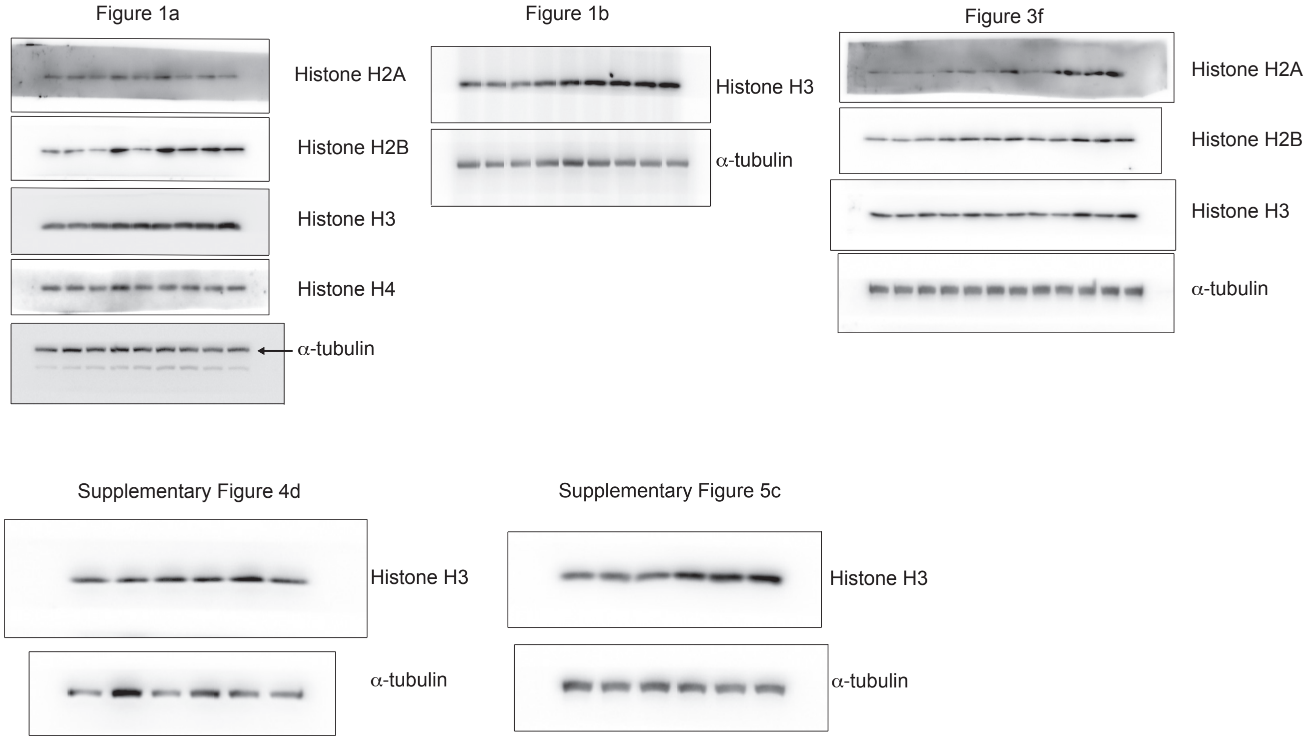

Supplementary Figure 6 Uncropped Western blots presented in this manuscript. Western blots were made by cutting the membrane based on molecular weight and incubating the cut slice with indicated antibody. Pictures, which were taken with Azure Western blot imaging system, show membrane slices from where the Western blot images were cropped.

Supplementary Table 1 Manually-curated genesets for GSEA.

Gene	WormBase ID	Gene	WormBase ID	Gene	WormBase ID
**HSP40, HSP60, HSP70 and HSP90 class heat shock proteins**		**Small heat shock proteins**		**Chromatin remodelers**	
dnj-1	WBGene00001019	*hsp-12.1*	WBGene00011906	*arp-1*	WBGene00007434
dnj-10	WBGene00001028	*hsp-12.2*	WBGene00002011	*C17E4.6*	WBGene00007645
dnj-12	WBGene00001030	*hsp-12.3*	WBGene00002012	*ekl-4*	WBGene00013676
dnj-13	WBGene00001031	*hsp-16.2*	WBGene00002016	*gfl-1*	WBGene00001585
dnj-19	WBGene00001037	*hsp-16.41*	WBGene00002018	*hsp-1*	WBGene00002005
F54F2.9	WBGene00018836	*hsp-16.48*	WBGene00002019	*htz-1*	WBGene00019947
hsp-60	WBGene00002025	*hsp-17*	WBGene00002021	*isw-1*	WBGene00002169
F44E5.5	WBGene00009692	*hsp-43*	WBGene00002024	*let-418*	WBGene00002637
F44E5.4	WBGene00009691	*sip-1*	WBGene00004798	*let-526*	WBGene00002717
F11F1.1	WBGene00008714	*Y55F3BR.6*	WBGene00021943	*lin-53*	WBGene00003036
hsp-1	WBGene00002005	*ZK1128.7*	WBGene00014233	*mep-1*	WBGene00003218
hsp-3	WBGene00002007			*mrg-1*	WBGene00003406
hsp-4	WBGene00002008			*nurf-1*	WBGene00009180
hsp-6	WBGene00002010			*pyp-1*	WBGene00008149
hsp-70	WBGene00002026			*rba-1*	WBGene00004312
hsp-110	WBGene00016250			*snfc-5*	WBGene00011111
stc-1	WBGene00006059			*ssl-1*	WBGene00007027
hsp-75	WBGene00020110			*swsn-1*	WBGene00004203
enpl-1	WBGene00011480			*swsn-2.2*	WBGene00015971
daf-21	WBGene00000915			*swsn-3*	WBGene00022182
				swsn-4	WBGene00004204
				swsn-6	WBGene00022774

				xnp-1	WBGene00006961
				zhit-1	WBGene00016992

Supplementary Table 2 Summary of *C. elegans* lifespan experiments.

Genotype, RNAi and treatment	mean lifespan ± SE (days)	variation compared to control (%)	*P*-values against control	N (trials)
**Figure 3b**				
N2, EV, RNAi regimen i	21.8 ± 0.36			104 (1)
N2, *isw-1* RNAi, RNAi regimen i	19.3 ± 0.2	- 11.5	$<10^{-3}$	110 (1)
N2, *mep-1* RNAi, RNAi regimen i	24.7 ± 0.46	+ 11.7	$<10^{-3}$	104 (1)
**Figure 3b**				
N2, EV, RNAi regimen i	20.1 ± 0.24			212 (2)
N2, *nurf-1* RNAi, RNAi regimen i	21.5 ± 0.26	+ 6.5	$<10^{-3}$	212 (2)
**Figure 3b**				
N2, EV, RNAi regimen i	21.3 ± 0.36			110 (1)
N2, *swsn-3* RNAi, RNAi regimen i	20.9 ± 0.34	- 1.9	0.3	109 (1)
**Figure 4a-b**				
N2, EV, RNAi regimen i	22.1 ± 0.35			202 (2)
N2, *isw-1* RNAi, RNAi regimen i	18.5 ± 0.1	-16.3	$<10^{-3}$	214 (2)
N2, EV, RNAi regimen ii	21.1 ± 0.55			90 (1)
N2, *isw-1* RNAi, RNAi regimen ii	21.2 ± 0.37	+ 0.5	0.43	95 (1)

**Figure 4c**				
N2, EV, RNAi regimen i	20.9 ± 0.28			219 (2)
N2, *isw-1* RNAi, RNAi regimen i	18.3 ± 0.1	- 12.4	<10^{-3}	227 (2)
N2, *isw-1* RNAi → EV, RNAi regimen iii	21.5 ± 0.27	+ 2.8	0.29	214 (2)
**Figure 5c**				
N2, EV	20.6 ± 0.2			432 (4)
isw-1 OE, EV	24.8 ± 0.24	+ 16.9	<10^{-3}	436 (4)
**Figure 5d**				
N2, EV	20.1 ± 0.21			317 (3)
N2, 4 sHSP RNAi	20.3 ± 0.9	+ 1	0.9	306 (3)
isw-1 OE, EV	24.7 ± 0.28	+ 18.6	<10^{-3}	307 (3)
isw-1 OE, 4 sHSP RNAi	22.5 ± 0.27	+ 10.7<br># - 8.9	<10^{-3}<br># <10^{-3}	309 (3)
**Figures 6a**				
N2, EV	21 ± 0.24			211 (2)
N2, *cco-1* RNAi	27.4 ± 0.36	+ 23.4	<10^{-3}	203 (2)
isw-1(n3297), EV	21 ± 0.21	0	0.21	183 (2)
isw-1(n3297), *cco-1* RNAi	23.2 ± 0.24	+ 9.5<br>¶ + 9.5	<10^{-3}<br>¶ <10^{-3}	213 (2)
**Figure 6b**				
N2, EV, RNAi regimen ii	20.6 ± 0.28			202 (2)
N2, EV/*cco-1* RNAi, RNAi regimen ii	26.4 ± 0.45	+ 22	<10^{-3}	215 (2)
N2, EV/*isw-1* RNAi, RNAi regimen ii	21.3 ± 0.2	+ 3.3	0.87	203 (2)
N2, *isw-1*/*cco-1* RNAi, RNAi regimen ii	23.5 ± 0.33	+ 12.3<br>*- 11	<10^{-3}<br>*<10^{-3}	224 (2)
**Figure 6c**				

N2, EV	20.0 ± 0.18			422 (4)
N2, *his-3* RNAi	21.4 ± 0.18	+ 6.5	$<10^{-3}$	414 (4)
**Figure 6d**				
isw-1(n3297), EV	19 ± 0.21			183 (2)
isw-1(n3297), *his-3* RNAi	18.2 ± 0.22	-4.2	0.09	196 (2)
**Figure 6e**				
N2, EV	22.8 ± 0.35			193 (2)
N2, EV/*his-3* RNAi	24.9 ± 0.42	+ 8.4	$<10^{-3}$	203 (2)
N2, EV/*cco-1* RNAi	29.2 ± 0.46	+ 21.9	$<10^{-3}$	212 (2)
N2, *his-3*/*cco-1* RNAi	28.6 ± 0.21	+ 20.3<br>^ - 2.1	$<10^{-3}$<br>^ 0.62	198 (2)
**Supplementary Figure 5a**				
N2, EV, RNAi regimen ii	22.0 ± 0.27			214 (2)
N2, *isw-1* RNAi, RNAi regimen ii	22.2 ± 0.21	+ 0.9	0.3	209 (2)
isw-1 OE, EV, RNAi regimen ii	24.8 ± 0.31	+ 11.3	$<10^{-3}$	198 (2)
isw-1 OE, *isw-1* RNAi, RNAi regimen ii	22.0 ± 0.18	+ 0<br>& - 11.3	0.03<br>& $<10^{-3}$	213 (2)
**Supplementary Figure 5e**				
hsf-1(sy441), EV	11 ± 0.22			231 (2)
hsf-1(sy441), *his-3* RNAi	10.6 ± 0.22	- 3.6	0.26	236 (2)

# Compared to *isw-1* OE, EV treatment

¶ Compared to *isw-1(n3297)*, EV treatment

* Compared to EV/*cco-1* RNAi treatment

^ Compared to EV/*cco-1* RNAi treatment

& Compared to *isw-1* OE, EV treatment

Supplementary Table 3. Sequences of oligonucleotides used for cloning in this study.

Region	Forward (5' → 3')	Reverse (5' → 3')
isw-1 promoter	CTAACATGTTGACCACATGCTTTCCAACG	CTAACCGGTTTTCTGTGACAATCACCAATTAACAAC
isw-1 ORF	CTAACCGGTATGTCGGTTCACGAGTCTTC	CTAGCTAGCTTATTTAGGAGTAGCTTTGACTTTCTTAGC
myo-2 promoter	ATAGCGTGCGGAGGTTTAGAG	GTAACCGGTTTCTGTGTCTGACGATCGAGG

Supplementary Table 4. Sequences of oligonucleotides used for qRT-PCR in this study.

Gene	Forward (5' → 3')	Reverse (5' → 3')
act-1	TCGGTATGGGACAGAAGGAC	CATCCCAGTTGGTGACGATA
pmp-3	GTTCCCGTGTTCATCACTCAT	ACACCGTCGAGAAGCTGTAGA
hsp-1	GACAAGAAGGGACACGGAGA	GATGAGTGTCTCCAGCGGTA
hsp-4	GACATCGAGCGCATGATCAA	CCTTGTCGGCGATTTGAGTT
hsp-6	AGAGCCAAGTTCGAGCAGAT	TCTTGAACAGTGGCTTGCAC
hsp-60	GGAAGCCCAAAGATCACAAA	CAGCCTCCTCATTAGCCTTG
hsp-12.1	AATTACAACTGACTCGGCGG	CGATTCCGTTCACCTTCACA
hsp-12.2	CAAGGTTTCCGGACAAGAGC	TGGAAGCAGTGATGGTGAGA
hsp-12.3	TGGACTTGAGGCTCACAACT	TGATGGTAGCTGGATCGGTT
hsp-12.6	GATTGGCCACTTCAAAAGGG	TCCTTCTTTACATTGTGCTCCA
hsp-16.1/ hsp-16.11	ACCACTATTTCCGTCCAGCT	ATCTTCTGGCTTGAACTGCG
hsp-16.2	AGATGTAGATGTTGGTGCAGT	TCTCTTCGACGATTGCCTGT
hsp-16.41	TGCTCCGTTCTCCATATTCTGA	AGAGACATCGAGTTGAACCGA
hsp-16.48/ hsp-16.49	CTCATGCTCCGTTCTCCATT	ACAATCTCTCCAATATTGTCGGA
rpn-1	TCTCTTTCCTCGACGCCAAT	GCTTGACCAACACGAACAGA
rpn-2	TGGAACAGGAAACATGGAAGC	GTCCTCGTTCTTCTCTCCGA
rpt-1	ATTGATGCTGTTGGAGGTGC	AGAGTGTCCGGTCTGTTTGT

rpt-2	GAGACAAGAAGAAGAGCGCG	AGAAGGACTGAACATCCCGG
ubq-1	CCTGGAGGTGGAGGCTTC	GTAGTCAGACAGGGTGCGG
ubq-2	TGGAGGAATCATCGAGCCAT	CCGCACTTCTTCTTTCTGCA
swsn-1	TCAGCAGCAGAGGGGATATG	TCCACCTTCATGATGTCCCC
swsn-3	GAATGGGAGGAGAAGCTTGC	CCGGCTTCTCCTTGACATTT
swsn-4	CTTCTCGTCCCTCAACCAGT	GCTTGATTTCCACCGGTCTG
swsn-8	CCGAGTGGCTGGAATGATTG	TGCAGCATCCGAGTTTGTAG
isw-1	GCAAAGTACAAGGCTCCGTT	TCTGAATTGTGGTGCCATGC
nurf-1	TGGGGAATCAACAACCGAGA	ACTTTGCGGATGTTGCTGTT
let-418	TTCTTTCGTTGAAGGTGCCG	TCAACAACAAGAGCTCCCCA
mep-1	TCAACCATCCAGCAAAAGCC	TCGCAAACTTGACTTGGTGG
mrg-1	GCATCATGATTGGAGTTCACGA	ATTGATTCGCTCCAACTCCG
atg-3	GTTCGGACCTATGATTTGCACA	CAGACGGATGAGCTTCAACAG
atg-7	TCCACTCAAGACACCAGCC	GCTGAAACTGTGCAGCGATA
atg-9	GGCCGCCATCCACTCATCGG	TTGACGTCGTGCCGCCGTAG
atg-11	TCGAGAATGGAGAGGAGCAC	TGCAAAACTTGGTCACCTCC
atg-18	AAATGGACATCGGCTCTTTG	TGATAGCATCGAACCATCCA
bec-1	CTGTCAGCATCCGTTGAGGT	AGAGCGTCAGAGCAATCATTACA
lgg-1	AGCACCAAAGTCAAAGCTCCA	CTTCCTCGTGATGGTCCTGG
dct-1	GCAAAAGCCGTCTCAAACCC	ACCCACGATTCTGACATACCA
sqst-1	GATCCTCCGACCACTCCAAA	TGGAAGTGGTGGAACGATCA
cco-1	GCTCGTCTTGCTGGAGATGATCGTT	GGTCGGCGTCGACTCCCTTG
H2A	GCCCCAAGACATCTTCAACT	GGAAGAAGAACAGCTTGGATG
H2B	CGTCTTGCTCACTACAACAAG	TTGGTTCCCTCAGACACGG
H3	TCTTCAAGAGGCTGCCGAG	AGCACGTTCTCCTCGGATAC
H4	TCGTGGAGTTCTCAAGGTGT	CCTCCGAATCCGTACAGAGT

htz-1	ATGGCTGGAGGAAAAGGAAA	GCTGGAAGTGGTTCTCTGCT
his-71	AGCTCCTCGCAAGCAGCT	GAAACAGCTCAACGTTTAT
his-72	CTTCCAGTCGGCTGCCAT	TCGAGAATTGGTGATGGAGC
hil-1	GAATTCCGGTTCAGACCAAA	TCCACGCTTTTTCATTGTTG
his-24	AAAGGTCCCAAAGGCTAAGG	ACTTGAGGATTGCCTGCTTG
hsp16.1/48 HRE1 ChIP-qRT-PCR	TCCTCTGAACACGATTGGCT	GCGTCTCTTTGCACCTATGG
hsp16.1/48 HRE2 ChIP-qRT-PCR	TTCTGAATGTGAGTCGCCCT	AGTGAATGTTGTTTGGTTCGGT
hsp16.2/41 HRE1 ChIP-qRT-PCR	TCTGAGCCCGCTTTCCTTAT	GCGTCTCTTTGCACCCAC
hsp16.2/41 HRE2 ChIP-qRT-PCR	TCCATGTACCGAATGTGAGTC	TGTTCGGTATTTATTTTCAACGGT
trt-1 ChIP-qRT-PCR	TCGGAGATGAAGCTGTCGG	CGGAGAGAAGAGGAGTACGG
